# Type of Androgen Deprivation Therapy and Risk of Dementia Among Patients With Prostate Cancer in Taiwan

**DOI:** 10.1001/jamanetworkopen.2020.15189

**Published:** 2020-08-31

**Authors:** Wen-Kuan Huang, Chi-Hung Liu, See-Tong Pang, Jia-Rou Liu, John Wen-Cheng Chang, Chuang-Chi Liaw, Cheng-Lung Hsu, Yung-Chang Lin, Lai-Chu See

**Affiliations:** 1 Division of Hematology and Oncology, Department of Internal Medicine, Chang Gung Memorial Hospital at Linkou, Chang Gung University College of Medicine, Taoyuan, Taiwan; 2 Department of Oncology-Pathology, Karolinska Institutet, Stockholm, Sweden; 3 Stroke Center, Department of Neurology, Chang Gung Memorial Hospital at Linkou, Chang Gung University College of Medicine, Taoyuan, Taiwan; 4 Division of Urology, Department of Surgery, Chang Gung Memorial Hospital at Linkou, Chang Gung University College of Medicine, Taoyuan, Taiwan; 5 Department of Public Health, Chang Gung University College of Medicine, Taoyuan, Taiwan; 6 Division of Rheumatology, Allergy, and Immunology, Department of Internal Medicine, Chang Gung Memorial Hospital at Linkou, Taoyuan, Taiwan; 7 Biostatistics Core Laboratory, Molecular Medicine Research Center, Chang Gung University, Taoyuan, Taiwan; 8 Center for Big Data Analytics and Statistics, Chang Gung Memorial Hospital at Linkou, Taoyuan, Taiwan

## Abstract

**Importance:**

It remains unclear whether androgen deprivation therapy (ADT) is associated with subsequent dementia risk in patients with prostate cancer. There are limited data regarding dementia risk across ADT types.

**Objective:**

To examine the association between all-cause dementia, including Alzheimer disease (AD), and different ADT types in patients with prostate cancer.

**Design, Setting, and Participants:**

This cohort study used linked data from the Taiwan National Cancer Registry, the National Health Insurance Research Database, and the Taiwan National Death Registry. A cohort of 23 651 patients with newly diagnosed prostate cancer between January 1, 2008, and December 31, 2015, was identified and followed up from 1 year after diagnosis until December 31, 2017. Data analysis was performed between January 2019 and May 2020.

**Exposures:**

Patients who received and did not receive ADT, including gonadotropin-releasing hormone (GnRH) agonists, orchiectomy, or antiandrogen monotherapy.

**Main Outcomes and Measures:**

The primary outcomes were all-cause dementia or AD. Stabilized inverse probability of treatment weighting was used to balance baseline covariates. The association between dementia and various ADT types was examined using the Cox proportional hazards model. Furthermore, a multivariate Cox proportional model with age as the time scale was conducted for complementary comparison.

**Results:**

In the cohort of 23 651 male patients (median [interquartile range] age, 73 [66-79] years), 6904 (29.2%) did not receive ADT, 11 817 (50.0%) received GnRH agonists, 876 (3.7%) received orchiectomy, and 4054 (17.1%) received antiandrogen monotherapy. Overall, 1525 patients were diagnosed with incident dementia (1.72 per 100 person-years) during a median (interquartile range) follow-up of 3.46 (1.92-5.51) years. Compared with those who did not receive ADT, those using antiandrogen monotherapy showed an increased risk of dementia (weighted hazard ratio [HR], 1.34; 95% CI, 1.16-1.55) and AD (weighted HR, 1.52; 95% CI, 1.13-2.04). The risk of dementia was similar between GnRH agonist use or orchiectomy and no ADT use (GnRH agonist: weighted HR, 1.13; 95% CI, 1.00-1.28; orchiectomy: 1.00; 95% CI, 0.74-1.37). Several sensitivity analyses revealed consistent findings for both outcomes.

**Conclusions and Relevance:**

In this study, the use of antiandrogen monotherapy was associated with increased risk of dementia or AD, while GnRH agonist use and orchiectomy had no significant difference compared with patients who did not receive ADT. Further prospective studies are warranted to confirm these findings.

## Key Points

**Question:**

Does the risk of dementia vary by type of androgen deprivation therapy among patients with prostate cancer?

**Findings:**

In this cohort study of 23 651 patients with newly diagnosed prostate cancer between 2008 and 2015, there was no association of the use of gonadotropin-releasing hormone agonists or orchiectomy with increased dementia risk, while antiandrogen monotherapy use was associated with a 34% increased dementia risk compared with patients who did not receive androgen deprivation therapy.

**Meaning:**

These findings suggest that the use of antiandrogen monotherapy, but not gonadotropin-releasing hormone agonist or orchiectomy, requires more attention with respect to subsequent risk of dementia.

## Teaser

This cohort study examines the association between all-cause dementia, including Alzheimer disease, and different types of androgen deprivation therapy among patients with prostate cancer in Taiwan.

## Introduction

Androgen deprivation therapy (ADT) is used with radiotherapy for patients with high-risk localized or locally advanced prostate cancer.^1^ Furthermore, ADT is the treatment mainstay for metastatic prostate cancer. Improved survival of patients with prostate cancer after ADT treatment has raised concerns regarding treatment-related adverse events, including decreased bone health and cardiometabolic aberrations.^2^ It remains unclear whether ADT use is associated with increased risk of dementia or Alzheimer disease (AD).^3,4^

Observational studies on the association between ADT use and dementia risk have reported inconsistent results.^5,6,7,8,9,10,11^ Two institution-based studies^7,8^ reported that ADT use was associated with increased risk of developing AD or all-cause dementia, which has drawn public attention to this issue. However, possible detection bias, immortal time bias, and inadequate statistical power to analyze dementia risk by ADT type limited the interpretation of these previous findings.^12^ Subsequent studies did not confirm this positive association.^9,10,11^ A large population-based study in the UK that used time-dependent exposure and robust methodology to reduce bias^9^ reported no association of ADT with dementia or AD.^9^ An analysis of 1.2 million Medicare beneficiaries with prostate cancer^10^ reported a clinically irrelevant risk of all-cause dementia (subdistribution hazard ratio [HR], 1.01; 95% CI, 1.01-1.02). In patients with nonmetastatic prostate cancer, receiving primary radiotherapy was not associated with dementia.^11^ These diverging results could be attributed to the heterogeneous patient populations, diverse data sources and analytic methods, and bias arising from study designs.

Even with androgen blockade through suppressed production or interrupting androgen receptor action, the effects on follicle-stimulating hormone (FSH) and luteinizing hormone (LH) levels differ between ADT types.^13,14^ For example, gonadotropin-releasing hormone (GnRH) antagonists decrease serum FSH and LH levels, while orchiectomy and antiandrogens increase their levels. Sustained use of GnRH agonist suppresses LH; however, FSH gradually increases over time. Furthermore, increased FSH and LH levels have been reported in men with dementia or AD.^15,16,17^ These previous findings suggest that the dementia risk may vary among ADT types. Consequently, we aimed to conduct a nationwide cohort study to examine the association between different ADT types and the risk of subsequent dementia, including AD, among patients with prostate cancer.

## Methods

### Data Sources

Patients with prostate cancer were identified from the Taiwan Cancer Registry Database, which contains details regarding individual characteristics at diagnosis; tumor pathology; TNM stage; primary treatment, including surgery, radiotherapy, and chemotherapy; and other systemic treatment. We linked the Taiwan Cancer Registry Database to the Taiwan National Health Insurance Research Database (NHIRD) and Taiwan National Death Registry. The NHIRD contains comprehensive and longitudinal claims data from approximately 99% of 23 million residents of Taiwan. This includes inpatient and outpatient information regarding date of visit, date of admission, procedures, prescription records, and diagnoses of major diseases with high accuracy according to the *International Classification of Diseases*, *Ninth Revision*, *Clinical Modification* (*ICD*-*9*-*CM*) and *ICD*-*10*-*CM* codes.^18,19^ The records of death in the National Death Registry have been validated.^20^ This study was approved by the institutional review board at Chang Gung Memorial Hospital. Because patient data had already been encrypted and deidentified, informed consent was not needed. This study is reported in accordance with the Strengthening the Reporting of Observational Studies in Epidemiology (STROBE) reporting guideline for cohort studies.

### Study Population

We included men aged at least 40 years with prostate cancer newly diagnosed between January 1, 2008, and December 31, 2015. We excluded patients with less than 1 year of follow-up after the diagnosis of prostate cancer or a history of dementia or AD and stroke or transient ischemic attack. Moreover, we excluded patients with a history of cancer before the diagnosis of prostate cancer or those who received both orchiectomy and GnRH agonist. Degarelix, a GnRH antagonist, has been reimbursed since September 2014 in Taiwan. We excluded patients who received a GnRH antagonist given the limited number of patients and the follow-up period. To exclude prevalent cases with a potential delay of diagnosis, the cohort entry was 1 year after the prostate cancer diagnosis. Patients were followed up after cohort entry until the incident diagnosis of dementia or AD, end of registration with National Health Insurance Program, death, or December 31, 2017 (end of the study period), whichever came first (eFigure in the Supplement).

### Exposure Assessment

We included the following ADTs: GnRH agonists (leuprolide, goserelin, triptorelin, and buserelin), bilateral orchiectomy, and oral antiandrogens (cyproterone acetate, bicalutamide, and flutamide). Using the prescription claim NHIRD database, we identified these drugs and categorized each patient into 1 of 4 mutually exclusive groups, as follows: did not receive ADT, received GnRH agonist, received orchiectomy, and received antiandrogen monotherapy. Antiandrogen use was allowed in the GnRH agonist or orchiectomy category if both FSH and LH levels on the combined application did not different from those on castration alone.^21^

### Covariates

Covariates known to be associated with dementia or AD were identified within 1 year before cohort entry. At least 2 consecutive records of diagnostic codes were required to confirm the comorbidity presence. In addition to age at diagnosis, which remains the greatest risk factor for dementia,^22^ we included cardiometabolic risk factors, including hypertension, diabetes, myocardial infarction, dyslipidemia, peripheral vascular disease, atrial fibrillation, and heart failure.^23^ Moreover, we included dementia-related comorbidities, including chronic medical illness, depression,^24^ traumatic head injury,^25^ hearing loss,^26^ and obstructive sleep apnea.^27^ Cancer stage and primary treatment were included as the tumor severity indexes. Stage information was based on the American Joint Committee on Cancer Sixth or Seventh edition. Urbanization level, monthly income, and enrollee category were used as proxies for socioeconomic status. Moreover, we included benzodiazepine^28^ and anticholinergic drugs,^29^ which are associated with dementia. We included anticholinergic drugs assigned as score 1 (possibly anticholingergic) or 2 (definitely anticholinergic) based on the 2012 update of the Anticholinergic Cognitive Burden Scale.^30^ Drug use was defined as using these medications for at least 6 months before cohort entry. eTable 1 and eTable 2 in the Supplement present the full list of diagnostic and drug codes, respectively. To minimize the detection bias resulting from patients receiving ADT having more medical visits, which increases the likelihood of recognizing incident dementia, we included the number of clinic visits within the first year after diagnosis of prostate cancer.

### Outcome Ascertainment

During the follow-up period, we identified all incident dementia cases, including AD. The diagnostic codes as primary diagnoses were recorded by a neurologist or a psychiatrist at least twice in outpatient visits or once in hospitalization (eTable 1 in the Supplement). In addition to the definitions using the AD diagnostic codes, patients who received galantamine, rivastigmine, donepezil, or memantine were defined as having incident AD. Taiwan National Health Insurance regulations require the reimbursement of these medications to patients after assessment by a neurologist or a psychiatrist using the Mini-Mental Status Examination or Clinical Dementia Rating, which have good diagnostic accuracy for AD.

### Statistical Analysis

Baseline characteristics were described as numbers (percentages) and medians (interquartile ranges [IQRs]). Absolute standardized mean differences (ASMD) were used to evaluate the balance between those who did not receive ADT and each group of patients who received ADT with a threshold of 0.1 indicating a significant imbalance.^31^ Since there were 4 study groups, the maximum pairwise ASMDs were reported. Crude incidence rates with 95% CIs were calculated by dividing the number of events by person-time at risk. For balanced comparison across the different treatment groups, we applied a stabilized inverse probability of treatment weighting (IPTW) for modeling causal outcomes using generalized boosting models.^32^ In this weighted analysis, we used the propensity score to generate stabilized weights, which preserved the original sample size and appropriately estimated the variance of the main outcome.^33^ All available covariates were contained in the generalized boosting model. Notably, cancer stage and primary treatment may inform the decision to select the ADT type; however, it is not directly associated with the outcome. Since adjusting for such variables increases bias and variance of the treatment-outcome estimate,^34^ they were not included as covariates for risk adjustment. We used a cause-specific hazard model to account for all-cause death as the competing risk because we intended to investigate the association between ADT use and incident dementia.^35^ The weighted cumulative incidence function curves for all-cause dementia and AD against years after prostate cancer diagnosis were plotted for all groups. Cox proportion hazards models were made with the inclusion of 1 variable (the 4 study groups) after stabilized IPTW. Furthermore, multivariate Cox proportion hazards models were conducted with age as the time scale and other covariates (urbanization level, monthly income, enrollee category, comorbidities, benzodiazepine, anticholingergic medications, and postdiagnosis clinical visit frequency per year) as the fixed scale. To reduce immortal time bias, we performed the time-dependent multivariate Cox regression model with ADT use as a time-dependent covariate and performed on-treatment analysis in which follow-up was censored at ADT discontinuation or change to other ADT types. Patients who did not receive ADT were the referent group in all analyses. A 2-sided α = .05 was the threshold for statistical significance. All analyses were performed using SAS version 9.4 (SAS Institute).

In sensitivity analyses, we excluded patients in the GnRH agonist group and orchiectomy group who received antiandrogens to allow assessment of the dementia risk among patients receiving pure ADT types. Moreover, we excluded patients who received postdiagnosis chemotherapy to account for the association of chemotherapy with cognitive impairment.

We performed subgroup analyses stratified with respect to age (≤70 vs >70 years), cardiometabolic risks (yes vs no), and cancer stage (I-III vs IV) to evaluate their association with dementia. For each subgroup analysis, a specific pseudopopulation was reassembled through stabilized IPTW to achieve balanced baseline covariates. We used interaction tests to assess the across-subgroup heterogeneity. We evaluated the duration-response association by analyzing cumulative duration of ADT use and dementia incidence. To estimate the strength of unmeasured confounders (ie, factors associated with both use of ADT and dementia) required to completely explain away the observed association, we calculated the E-value proposed by Vanderweele.^36^

## Results

### Patient Characteristics

The database extracted for the current analysis comprised 23 651 patients with newly diagnosed prostate cancer (median [IQR] age, 72 [66-79] years) who met the inclusion criteria (eFigure in the Supplement). Among them, 6904 (29.2%) did not receive ADT, 11 817 (50.0%) received GnRH agonists, 876 (3.7%) received orchiectomy, and 4054 (17.1%) received antiandrogen monotherapy. The Table presents the baseline patient characteristics stratified by ADT types. Patients who did not receive ADT were younger than those who did (median [IQR] age, no ADT: 68 [63-74] years; GnRH agonist, 74 [67-80] years; orchiectomy, 75 [69-81] years; antiandrogen monotherapy, 75 [68-80] years). Recipients of GnRH agonist or orchiectomy had more advanced diseases than those who received antiandrogen monotherapy or did not receive ADT (stage IV disease among GnRH group: 5255 [44.5%]; orchiectomy group: 502 [57.3%]; antiandrogen group, 258 [6.4%]; no ADT group, 109 [1.6%]). Antiandrogen was mostly used for early-stage disease (stages I and II, 2714 [66.9%]). There was no significant among-group difference in the prevalence of each comorbidity. After weighting, a considerable across-group balance of baseline covariates was achieved.

**Table.** Baseline Characteristics of Patients With Prostate Cancer in the 4 Study Groups

Characteristic	Before weighting					After weighting				
	Patients, No. (%)				Maximum ASMD^a^	Patients, No. (%)				Maximum ASMD^a^
	No ADT (n = 6904)	GnRH agonist (n = 11 817)	Orchiectomy (n = 876)	Antiandrogen monotherapy (n = 4054)		No ADT (n = 6904)	GnRH agonist (n = 11 817)	Orchiectomy (n = 876)	Antiandrogen monotherapy (n = 4054)	
Age at diagnosis, y										
Median (IQR)	68 (63-74)	74 (67-80)	75 (69-81)	75 (68-80)	NA	75 (66-78)	72 (66-79)	73 (66-79)	72 (66-79)	NA
40-59	951 (13.8)	878 (7.4)	35 (4.0)	242 (6.0)	0.6892	592 (8.8)	1024 (8.8)	42 (5.5)	332 (8.5)	0.1161
60-69	2904 (42.1)	3030 (25.6)	204 (23.3)	917 (22.6)		2039 (30.3)	3469 (29.7)	231 (30.5)	1154 (29.4)	
70-79	2288 (33.1)	4920 (41.6)	377 (43.0)	1741 (43.0)		2671 (39.7)	4627 (39.6)	321 (42.3)	1581 (40.3)	
≥80	761 (11.0)	2989 (25.3)	260 (29.7)	1154 (28.5)		1430 (21.3)	2561 (21.9)	164 (21.7)	860 (21.9)	
Urbanization										
Urban area	2111 (30.6)	3074 (26.0)	190 (21.7)	1251 (30.9)	0.2450	1898 (28.2)	3260 (27.9)	193 (25.5)	1105 (28.1)	0.0870
Suburban area	1936 (28.0)	3133 (26.5)	239 (27.3)	1083 (26.7)		1811 (26.9)	3141 (26.9)	202 (26.7)	1066 (27.2)	
Rural area	2685 (38.9)	5274 (44.6)	416 (47.5)	1612 (39.8)		2842 (42.2)	4958 (42.5)	342 (45.2)	1651 (42.0)	
Not specified	172 (2.5)	336 (2.8)	31 (3.5)	108 (2.7)		181 (2.7)	322 (2.8)	20 (2.7)	105 (2.7)	
Monthly income, NT$^b^										
Dependent	2605 (37.7)	4504 (38.1)	296 (33.8)	1458 (36.0)	0.4143	2505 (37.2)	4379 (37.5)	303 (40.0)	1460 (37.2)	0.1604
<15 000	1779 (25.8)	3102 (26.3)	289 (33.0)	1308 (32.3)		1857 (27.6)	3189 (27.3)	205 (27.0)	1080 (27.5)	
15 000-24 999	1598 (23.2)	3426 (29.0)	267 (30.5)	1024 (25.3)		1803 (26.8)	3134 (26.8)	212 (28.0)	1065 (27.1)	
≥25 000	922 (13.4)	785 (6.6)	24 (2.7)	264 (6.5)		567 (8.4)	979 (8.4)	38 (5.0)	323 (8.2)	
Enrollee category^c^										
1	563 (8.2)	821 (7.0)	52 (5.9)	276 (6.8)	0.3590	481 (7.1)	836 (7.2)	55 (7.2)	283 (7.2)	0.0662
2	2110 (30.6)	2771 (23.5)	148 (16.9)	967 (23.9)		1729 (25.7)	2958 (25.3)	182 (24.0)	1004 (25.6)	
3	2145 (31.1)	4618 (39.1)	354 (40.4)	1350 (33.3)		2382 (35.4)	4210 (36.0)	288 (38.0)	1406 (35.8)	
4	2086 (30.2)	3607 (30.5)	322 (36.8)	1461 (36.0)		2140 (31.8)	3677 (31.5)	233 (30.7)	1235 (31.4)	
Stage										
I	1230 (17.8)	231 (2.0)	5 (0.6)	376 (9.3)	1.7100	1241 (18.4)	236 (2.0)	6 (0.8)	375 (9.5)	1.7699
II	3797 (55.0)	3000 (25.4)	164 (18.7)	2338 (57.7)		3713 (55.2)	2973 (25.5)	131 (17.3)	2227 (56.7)	
III	899 (13.0)	2110 (17.9)	122 (13.9)	578 (14.3)		810 (12.0)	2149 (18.4)	118 (15.6)	593 (15.1)	
IV	109 (1.6)	5255 (44.5)	502 (57.3)	258 (6.4)		135 (2.0)	5084 (43.5)	435 (57.4)	241 (6.1)	
Unknown	869 (12.6)	1221 (10.3)	83 (9.5)	504 (12.4)		832 (12.4)	1238 (10.6)	68 (8.9)	491 (12.5)	
Primary treatment										
Prostatectomy	4920 (71.3)	3571 (30.2)	261 (29.8)	1763 (43.5)	0.9215	4513 (67.0)	3611 (30.9)	209 (27.6)	1806 (46.0)	0.8620
Radiotherapy	809 (11.7)	4612 (39.0)	298 (34.0)	1353 (33.4)		945 (14.0)	4586 (39.3)	287 (37.9)	1294 (32.9)	
Other or unknown	1175 (17.0)	3634 (30.8)	317 (36.2)	938 (23.1)		1274 (18.9)	3484 (29.8)	261 (34.5)	828 (21.1)	
Myocardial infarction	41 (0.6)	86 (0.7)	8 (0.9)	27 (0.7)	0.0369	46 (0.7)	77 (0.7)	3 (0.4)	24 (0.6)	0.0369
Coronary heart disease	379 (5.5)	629 (5.3)	65 (7.4)	261 (6.4)	0.0859	374 (5.6)	641 (5.5)	40 (5.3)	220 (5.6)	0.0142
Heart failure	69 (1.0)	180 (1.5)	18 (2.1)	76 (1.9)	0.0861	89 (1.3)	163 (1.4)	10 (1.4)	52 (1.3)	0.0073
Peripheral vascular disease	16 (0.2)	38 (0.3)	3 (0.3)	16 (0.4)	0.0292	18 (0.3)	35 (0.3)	1 (0.2)	11 (0.3)	0.0351
COPD	151 (2.2)	365 (3.1)	36 (4.1)	162 (4.0)	0.1102	203 (3.0)	349 (3.0)	23 (3.0)	118 (3.0)	0.0017
Atrial fibrillation	146 (2.1)	273 (2.3)	23 (2.6)	116 (2.9)	0.0479	149 (2.2)	268 (2.3)	13 (1.8)	91 (2.3)	0.0417
Hypertension	3373 (48.9)	5960 (50.4)	437 (49.9)	2181 (53.8)	0.0990	3428 (50.9)	5885 (50.4)	378 (49.9)	1995 (50.8)	0.0220
Diabetes	1392 (20.2)	2617 (22.2)	163 (18.6)	909 (22.4)	0.0945	1451 (21.6)	2516 (21.5)	155 (20.5)	844 (21.5)	0.0266
Dyslipidemia	1672 (24.2)	2425 (20.5)	136 (15.5)	879 (21.7)	0.2191	1458 (21.7)	2503 (21.4)	151 (20.0)	838 (21.4)	0.0437
Traumatic head injury	429 (6.2)	995 (8.4)	63 (7.2)	348 (8.6)	0.0906	514 (7.6)	906 (7.8)	49 (6.4)	307 (7.8)	0.0557
Depression	685 (9.9)	1032 (8.7)	79 (9.0)	423 (10.4)	0.0578	624 (9.3)	1076 (9.2)	67 (8.8)	368 (9.4)	0.0212
Hearing loss	809 (11.7)	1346 (11.4)	111 (12.7)	558 (13.8)	0.0716	810 (12.0)	1386 (11.9)	90 (11.9)	466 (11.9)	0.0053
Sleep apnea	246 (3.6)	242 (2.1)	12 (1.4)	92 (2.3)	0.1417	169 (2.5)	280 (2.4)	12 (1.6)	94 (2.4)	0.0678
Peptic ulcer disease	859 (12.4)	1509 (12.8)	125 (14.3)	546 (13.5)	0.0537	863 (12.8)	1483 (12.7)	103 (13.5)	491 (12.5)	0.0321
Chronic liver disease	742 (10.8)	1098 (9.3)	82 (9.4)	432 (10.7)	0.0485	658 (9.8)	1153 (9.9)	75 (9.9)	391 (10.0)	0.0061
Chronic kidney disease	769 (11.1)	1736 (14.7)	116 (13.2)	532 (13.1)	0.1061	880 (13.1)	1555 (13.3)	104 (13.7)	506 (12.9)	0.0249
Rheumatic diseases	431 (6.2)	660 (5.6)	34 (3.9)	256 (6.3)	0.1108	397 (5.9)	670 (5.7)	34 (4.5)	229 (5.8)	0.0646
Medications with >6 mo use^d^										
Benzodiazepine	2099 (30.4)	3742 (31.7)	259 (29.6)	1415 (34.9)	0.1144	2166 (32.2)	3702 (31.7)	235 (31.0)	1255 (32.0)	0.0257
Anticholinergic	3733 (54.1)	6386 (54.0)	440 (50.2)	2426 (59.8)	0.1941	3724 (55.3)	6403 (54.8)	415 (54.8)	2172 (55.3)	0.0107
Clinical visits, No.^e^										
Median (IQR)	38 (27-53)	46 (37-65)	45 (33-61)	47 (34-63)	NA	41 (29-59)	45 (33-60)	45 (33-61)	44 (32-60)	NA
0-12	282 (4.1)	106 (0.9)	19 (2.2)	53 (1.3)	0.3627	133 (2.0)	210 (1.8)	12 (1.6)	66 (1.7)	0.0212
13-52	4890 (70.8)	7194 (60.9)	539 (61.5)	2374 (58.6)		4311 (64.0)	7412 (63.5)	476 (62.9)	2489 (63.4)	
53-high	1732 (25.1)	4517 (38.2)	318 (36.3)	1627 (40.1)		2288 (34.0)	4058 (34.7)	270 (35.6)	1373 (35.0)	

Abbreviations: ADT, androgen deprivation therapy; ASMD, absolute standardized mean difference; COPD, chronic obstructive pulmonary disease; GnRH, gonadotropin-releasing hormone; IQR, interquartile range; NA, not applicable; NT$, new Taiwan dollar.

^a^ Maximum of pairwise absolute standardized mean difference ≤0.1 indicates a negligible difference.

^b^ To convert NT$ to US dollars, multiply by 0.034.

^c^ Enrollee category 1 indicates civil servants, full-time or regularly paid personnel in governmental agencies and public schools; 2, employees of privately owned enterprises or institutions; 3, self-employed, other employee, or paid personnel and members of the farmers or fishers associations; 4, members of low-income families, military service, and veterans.

^d^ Before cohort entry, which was defined as 1 year after the diagnosis of prostate cancer.

^e^ The number of clinical visits was counted within the first year after diagnosis of prostate cancer.

### Adjusted Risk of Dementia and AD

**Figure 1.**
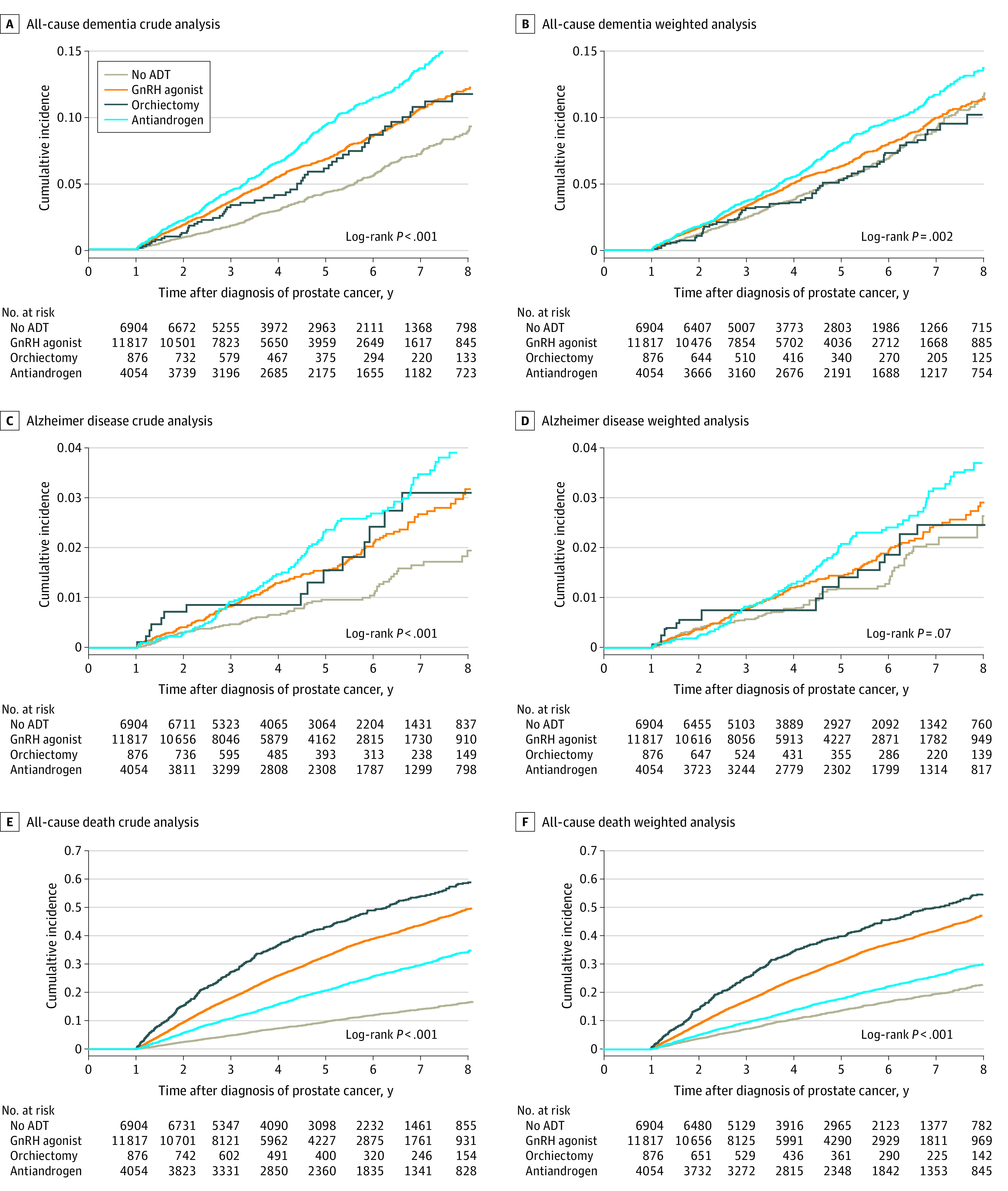
Crude and Weighted Cumulative Incidence for Dementia and All-Cause Death by Androgen Deprivation Therapy (ADT) Type GnRH indicates gonadotropin-releasing hormone.

Overall, within the median (IQR) follow-up period of 3.46 (1.92-5.51), the crude incidence rates of all-cause dementia and AD per 100 person-years in the entire cohort were 1.76 (95% CI, 1.67-1.85) per 100 person-years and 0.41 (95% CI, 0.37-0.45) per 100 person-years, respectively. Figure 1 presents the crude and weighted cumulative incidences for all-cause dementia, AD, and all-cause death across the 4 study groups.

**Figure 2.**
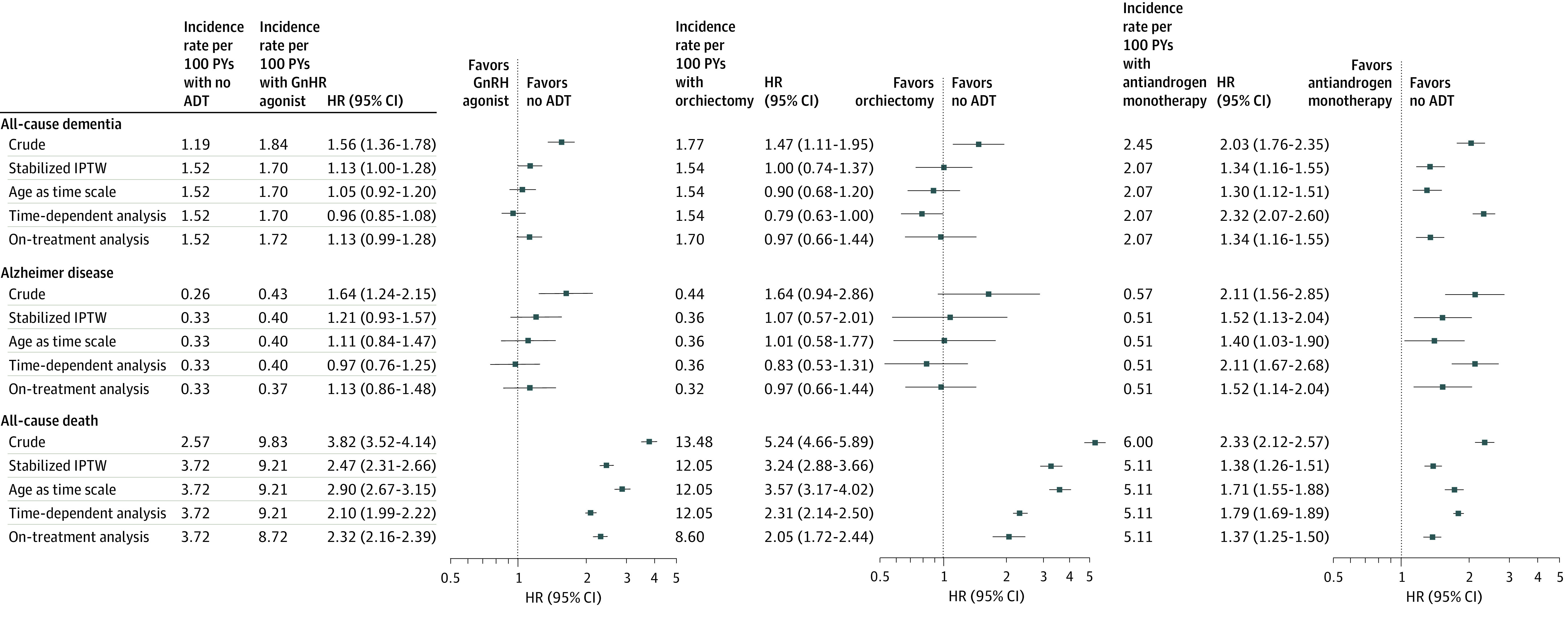
Incidence Rates and Hazard Ratios (HRs) for Dementia and All-Cause Death by Androgen Deprivation Therapy (ADT) Type GnRH indicates gonadotropin-releasing hormone; IPTW, inverse probability of treatment weighting.

In the stabilized IPTW-adjusted Cox proportional hazard regression model analyses, compared with those who did not receive ADT, antiandrogen monotherapy use was associated with an increased risk of all-cause dementia (HR, 1.34; 95% CI, 1.16-1.55) and AD (HR, 1.52; 95% CI, 1.13-2.04). The major findings were consistent with analyses using multivariate Cox proportion hazards models with age as the time scale (Figure 2). Compared with those who did not receive ADT, there was no significant difference in the risk of dementia or AD among patients treated with GnRH agonist or orchiectomy (GnRH agonist: weighted HR, 1.13; 95% CI, 1.00-1.28; orchiectomy: 1.00; 95% CI, 0.74-1.37). Time-dependent multivariate Cox regression model and on-treatment analysis yielded similar results, suggesting a minimal effect of immortal time bias (Figure 2).

### Sensitivity and Subgroup and Analyses

**Figure 3.**
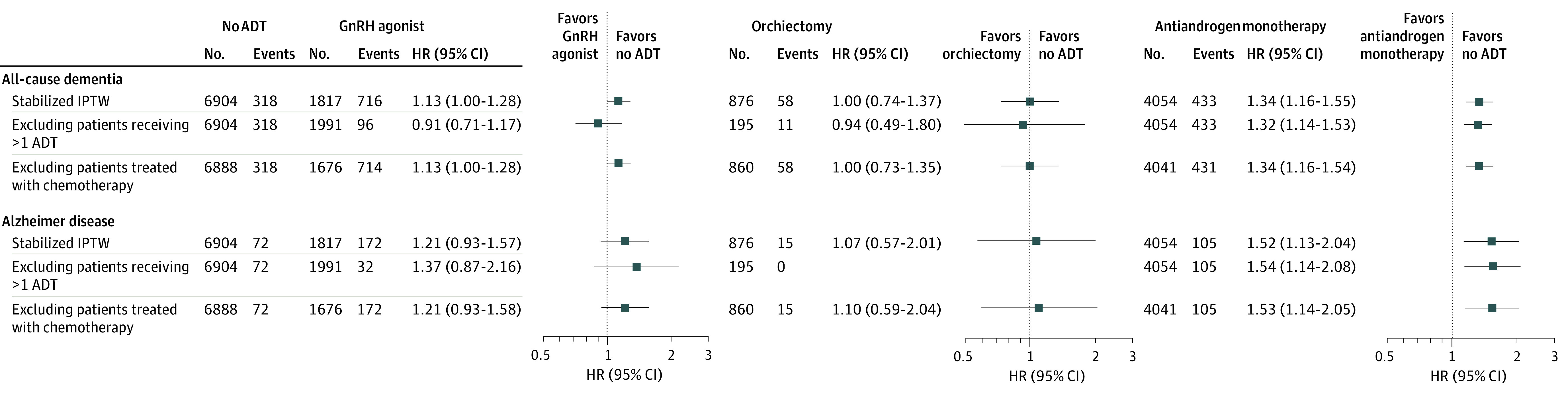
Sensitivity Analysis to Assess the Association Between Different Types of Androgen Deprivation Therapy (ADT) and Dementia GnRH indicates gonadotropin-releasing hormone; HR, hazard ratio; IPTW, inverse probability of treatment weighting.

Exclusion of patients in the GnRH agonist and orchiectomy groups who received antiandrogen treatment yielded an increased risk of all-cause dementia (HR, 1.32; 95% CI, 1.14-1.53) and AD (HR, 1.54; 95% CI, 1.14-2.08), which was consistent with the main results (Figure 3). Another sensitivity analysis revealed that there was no significant change with the exclusion of patients treated with chemotherapy (Figure 3).

**Figure 4.**
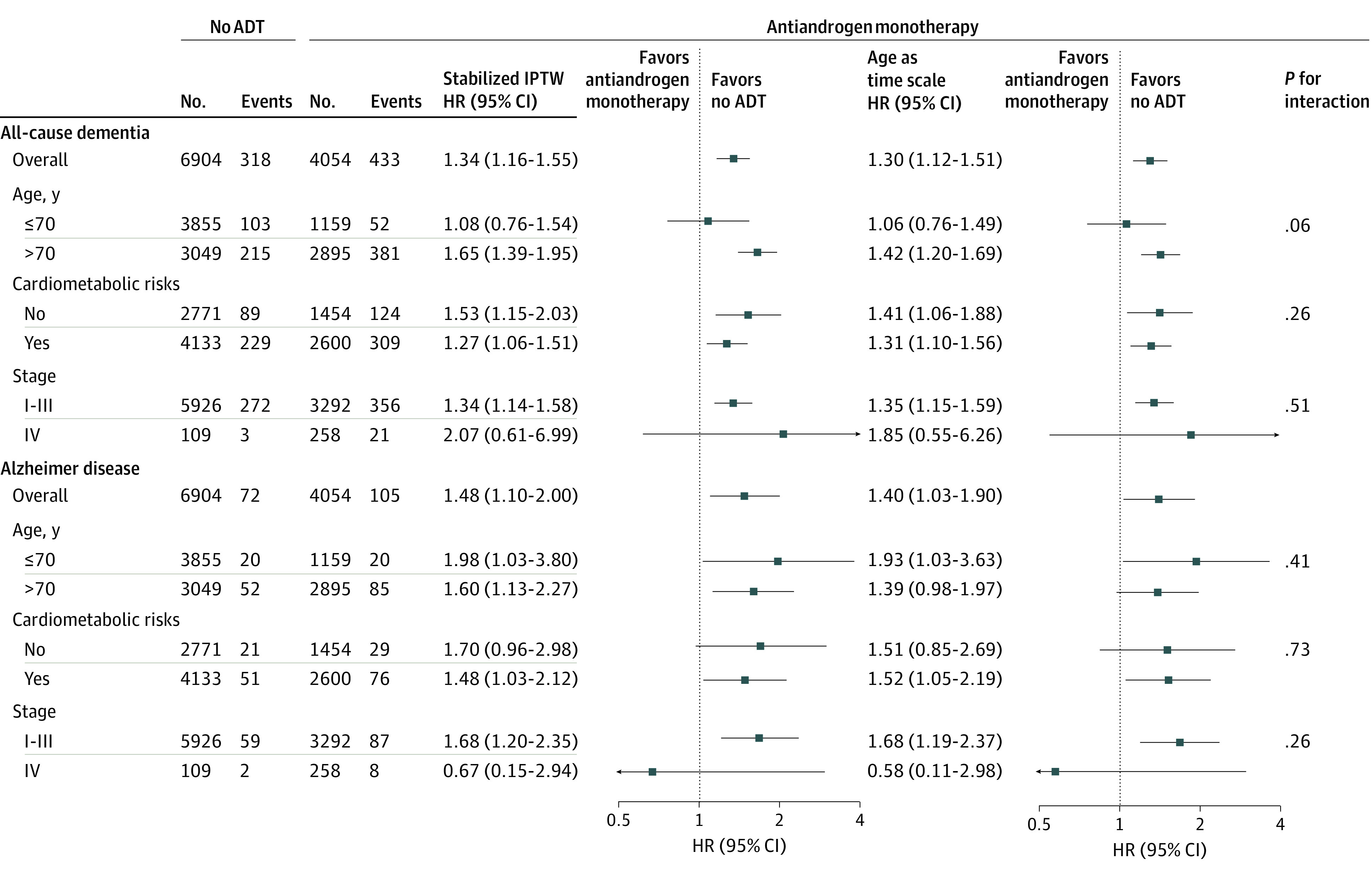
Subgroup Analysis for Dementia Risk of Antiandrogen Monotherapy ADT indicates androgen deprivation therapy; HR, hazard ratio.

Figure 4 summarizes the results of subgroup analyses. The association of antiandrogen monotherapy use with an increased risk of all-cause dementia was consistent across subgroups. Duration-response analyses revealed an increased dementia risk with a treatment duration of less than 2 years (eTable 3 in the Supplement), which subsequently decreased. The E-value for the association between antiandrogen monotherapy and dementia was 2.01, with a lower bound of 1.59, which indicates that an unmeasured confounder required a strong association with antiandrogen monotherapy use and dementia by an HR of 2.01-fold each to explain away the observed association.

## Discussion

In this cohort of more than 20 000 men with prostate cancer, antiandrogen monotherapy use was associated with an increased risk of all-cause dementia or AD compared with those who did not receive ADT. There was no significant difference in the risk of either dementia or AD between patients receiving GnRH agonist or orchiectomy and those not receiving ADT. These results contribute to the critical assessment of dementia risk according to ADT types.

Only a few studies on the association between ADT and dementia have reported the dementia risk according to ADT type.^9,37^ Khosrow-Khavar et al^9^ reported similar dementia risks of GnRH agonist or antiandrogen monotherapy compared with those who did not receive ADT. Notably, the overall incidence of dementia in their study (approximately 0.59 per 100 person-years)^9^ was significantly lower than that reported by Baik et al^10^ (3.27 per 100 person-years) and our study (1.76 per 100 person-years). Baik et al^10^ reported a very small increased risk (1%) of dementia and a small decreased risk (2%) of AD after ADT treatment based on a cohort of 1.2 million patients from a set of Medicare beneficiaries. However, the lack of data regarding the use of antiandrogen and the small proportion of orchiectomy recipients (1.1%) limited the dementia risk estimation across different ADT types. Two previous studies by Nead et al^7,8^ had limited power for analyzing the dementia risk by ADT type. A 2019 Swedish cohort study reported that GnRH agonist or orchiectomy use, but not antiandrogen monotherapy use, was associated increased dementia risks. Antiandrogen monotherapy was mostly used for early-stage and advanced-stage prostate cancer in our cohort (66.9%) and the Swedish cohort (71.3%), respectively, which suggests differences with respect to baseline characteristics and treatment duration. This could be partly attributed to differences in the practice patterns of ADT between the Swedish study and our study.

In our study, there was increased risk during a limited period after antiandrogen treatment initiation. However, there was no sustained increased risk with longer treatment duration. This indicates that ADT may exacerbate the preexisting neurodegeneration process. There is a need for future studies with larger sample sizes to assess dementia on ADT treatment.

The mechanism underlying the ADT causes of dementia remains unclear and could be multifactorial. Studies have reported an association of reduced testosterone level with AD and dementia.^38,39^ Moreover, epidemiological and in vivo studies have suggested that LH contributes to cognitive decline and β-amyloid accumulation, which is a pathological AD hallmark.^40,41^ We did not identify a clinically meaningful hazard on GnRH agonist treatment compared with patients who did not receive ADT. GnRH agonist usage reduces testosterone and LH levels. Future studies should assess whether the suppressed LH counteracts the biological effect of low testosterone on cognitive decline. We speculated that increased LH level on treatment using antiandrogen monotherapy could partly explain the association of antiandrogen monotherapy with dementia and AD. There is a need for further studies to elucidate the underlying mechanisms of the association between antiandrogen and dementia.

### Strengths and Limitations

We analyzed a nationwide population, which was adjusted extensively for dementia-related comorbidities and medications, and preformed multiple subgroup and sensitivity analyses to assess the robustness of the main findings. In contrast to previous studies,^7,8,9^ we performed subgroup analyses to account for tumor stage, which improves the strength of this study. However, this study has several limitations. First, dementia or AD events recorded using diagnostic codes in the claim data may be inaccurate. However, we examined patient records from more than 2 clinic visits by specialists rather than general practitioners to increase diagnostic validity. Furthermore, the incidence rate observed in our study was similar to that observed in a Taiwanese study using the Mini-Mental Status Examination.^42^ Second, given this study’s observational nature and inherent NHIRD limitations, we could not account for residual confounding effects from unmeasured variables, including family history, education level, smoking status, and physical inactivity. Our calculated E-value of 2.01 showed that an unmeasured confounder would have to be at this magnitude or greater with both antiandrogen treatment and dementia to explain away our findings. The presence of such a confounder is unlikely because the range of point estimates for the HRs for all included risk factors were lower than the calculated E-value. Furthermore, there is a risk of detection bias because of the longer follow-up period while differences in the medical checkup frequency between those who did and did not receive ADT was expected to decrease over time.

## Conclusions

This cohort study found an association between antiandrogen monotherapy and the risk of all-cause dementia or AD. However, the use of GnRH agonist or orchiectomy was not associated with increased risk of dementia or AD. There is a need for future prospective studies to confirm these results.

## Notes

Supplement. **eFigure. **Study Flow Diagram **eTable 1. **Codes for Comorbidities and Outcomes **eTable 2. **Anatomical-Therapeutic-Chemical (ATC) Codes for Medications **eTable 3. **Crude and Adjusted Hazard Ratios for the Association Between Antiandrogen Monotherapy and All-Cause Dementia by Cumulative Use After Prostate Cancer Diagnosis
