# Supplementary material for: Type of Androgen Deprivation Therapy and Risk of Dementia Among Patients With Prostate Cancer in Taiwan
Source: JAMA Netw Open. 2020 Aug 31;3(8):e2015189. doi: 10.1001/jamanetworkopen.2020.15189 (PMC7489824; doi:10.1001/jamanetworkopen.2020.15189)
Supplement: Supplement. — eFigure. Study Flow Diagram eTable 1. Codes for Comorbidities and Outcomes eTable 2. Anatomical-Therapeutic-Chemical (ATC) Codes for Medications eTable 3. Crude and Adjusted Hazard Ratios for the Association Between Antiandrogen Monotherapy and All-Cause Dementia by Cumulative Use After Prostate Cancer Diagnosis [file jamanetwopen-e2015189-s001.pdf]

Supplementary Online Content

Huang W-K, Liu C-H, Pang S-T, et al. Type of androgen deprivation therapy and risk of dementia among patients with prostate cancer in Taiwan. *JAMA Netw Open*. 2020;3(8):e2015189. doi:10.1001/jamanetworkopen.2020.15189

eFigure. Study Flow Diagram
eTable 1. Codes for Comorbidities and Outcomes
eTable 2. Anatomical-Therapeutic-Chemical (ATC) Codes for Medications
eTable 3. Crude and Adjusted Hazard Ratios for the Association Between Antiandrogen Monotherapy and All-Cause Dementia by Cumulative Use After Prostate Cancer Diagnosis

This supplementary material has been provided by the authors to give readers additional information about their work.

© 2020 Huang W-K et al. *JAMA Network Open*.

eFigure. Study Flow Diagram

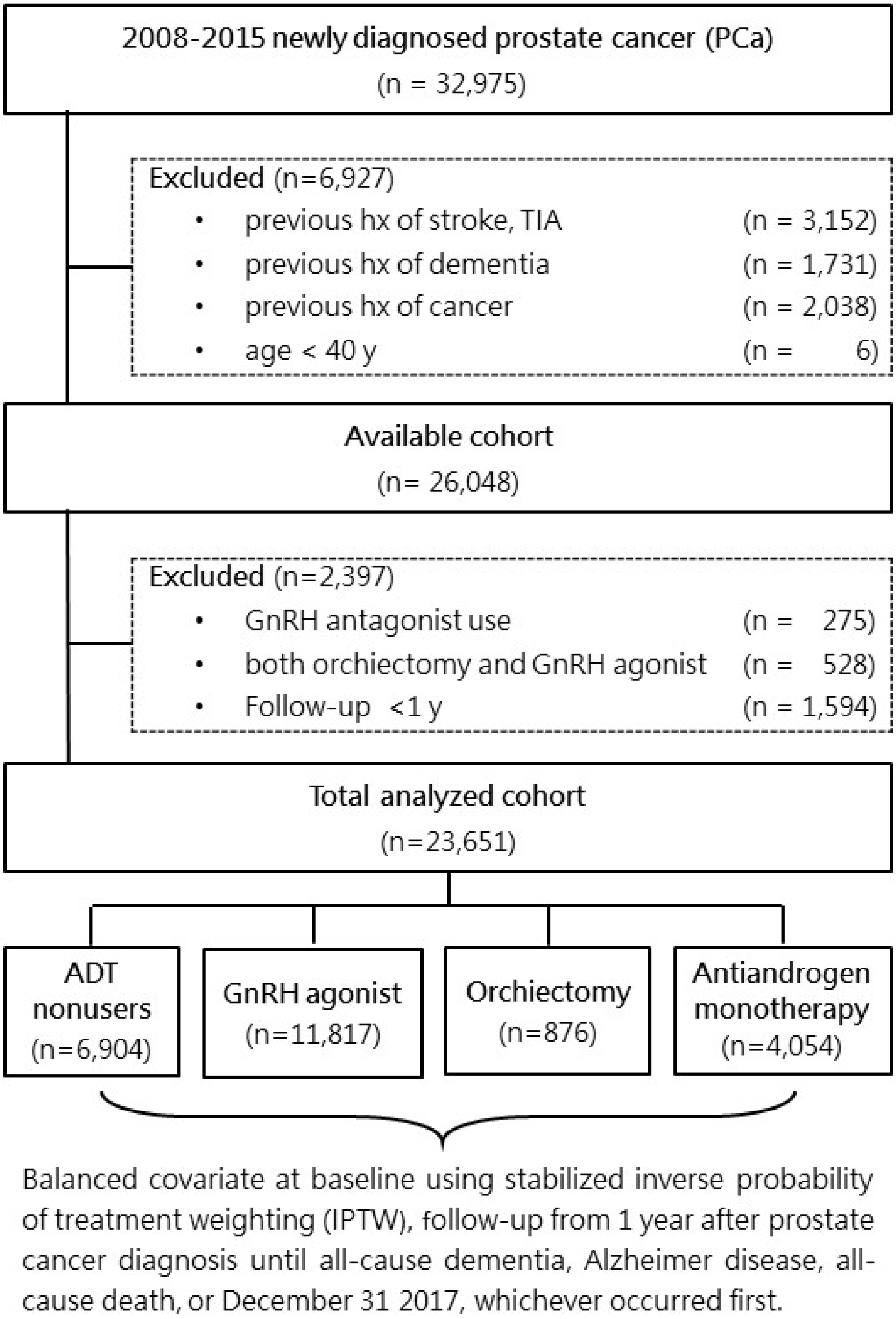

© 2020 Huang W-K et al. *JAMA Network Open*.

eTable 1. Codes for Comorbidities and Outcomes

	International Classification of Disease 9th revision (ICD-9) code	International Classification of Disease 10th revision (ICD-10) code
**Comorbidities**		
Myocardial infarction	410, 412	I21, I25.2
Coronary heart disease	411-414	I20, I24, I25
Heart failure	428	I50
Peripheral vascular disease	437.3, 440, 441, 443, 447.1, 785.4	I67.1, I70, I71, I73, I77.1, I77.7, I96
Chronic obstructive pulmonary disease	490-492, 493.2, 496	J40-J44
Atrial fibrillation	427.31	I48.91
Hypertension	401-404	I10-I13
Diabetes mellitus	250	E10-E11
Dyslipidemia	272	E75.2, E77.0, E77.1, E78, E88.89
Traumatic head injury	800-804, 850-854	S02, S06
Depression	296.2, 296.3, 300.4, 311	F32, F33, F34.1
Hearing loss	389	H90, H91.3, H91.8, H91.9
Sleep apnea	327.23, 780.51, 780.53, 780.57	G47.33, G47.30
Peptic ulcer disease	531-534	K25-K28
Chronic liver disease	570, 571, 573.3, 573.4, 573.8, 573.9, 456.0-456.2, 572.2-572.8	K70, K71.6, K72, K73, K76, I85.0, I85.1
Chronic kidney disease	250.4, 271.4, 274.1, 403, 404, 440.1, 442.1, 446.21, 447.3, 572.4, 580-589, 590, 591, 593	E10-E11, E74.8, M10.3, N20.0, M10.3, I12, I13.1, I70.1, I72.2, M31.0, I77.3, K76.7, N00-N05, N10-N12, N13.30, N15-N19, N28
Rheumatic diseases	446.5, 710.0-710.4, 714.0-714.2, 714.8, 714.9, 725	M05.0, M05.3, M05.6, M05.10, M35.3, M31.6, M34.0, M34.1, M34.9, M06.1, M06.4, M06.9, M32.10, M33.20, M33.90, M35.0
**Outcomes**		
All-cause dementia	290.0-290.4, 294.1, 331.0-331.2, 331.82, 438.0	F01.50, F01.51, F02.81, F03.9, F05, G30.9, G31.01, G31.09, G31.1, G31.83, I69.91
Alzheimer's disease	331.0	G30.9

© 2020 Huang W-K et al. *JAMA Network Open*.

eTable 2. Anatomical-Therapeutic-Chemical (ATC) Codes for Medications

**Media ions**	**ATC codes**
GnRH agonist	
Leuprolide	L02AE02
Goserelin	L02AE03
Triptorelin	L02AE04
Buserelin	L02AE01
Anti-androgen	
Cyproterone	G03HA01, G03HB01
Bicalutamide	L02BB03
Flutamide	L02BB01
GnRH antagonist	
Degarelix	L02BX02
Benzodiazepine, BZD	N03AE01, N05BA01, N05BA02, N05BA03, N05BA04, N05BA05, N05BA06, N05BA08, N05BA09, N05BA11, N05BA12, N05BA16, N05BA17, N05BA22, N05BA91, N05CD01, N05CD02, N05CD03, N05CD04, N05CD05, N05CD06, N05CD07, N05CD08, N05CD09, N05CD91
Anti-cholinergic	A02AG, A02AX, A02X, A03AA07, A03AB05, A03AB20, A03BA01, A03BA03, A03BA91, A03BB01, A03BB02, A03BB03, A03CA01, A03CB, A03CB02, A03CB31, A03CC, A03DB04, A03ED, A04AD01, A07AX, A09AA, C05AD03, D02AB, D04AA32, D04AB04, D04AX91, D07BA01, D07XA01, D07XA02, G01AX, G04BD02, G04BD04, G04BD06, G04BD07, G04BD08, G04BD09, J05AX, M02AC, M03BA03, M03BC01, M03BC51, M03BX08, N02AA51, N02AB02, N02AG, N02BA07, N02BB52, N02BB72, N02BE51, N02BG06, N02CA52, N02CA72, N03AA, N03AF01, N03AF02, N04AA01, N04AC01, N04BB01, N05AA, N05AA01, N05AA02, N05AB03, N05AB06, N05AC02, N05AG02, N05AH01, N05AH02, N05AH03, N05AH04, N05BB01, N06AA02, N06AA04, N06AA09, N06AA12, N06AB05, N06CA01, R01AB02, R01AX30, R01BA51, R01BA52, R01BA53, R03CA, R03CB53, R03CK, R03DA05, R03DA51, R03DA55, R03DA71, R03DA75, R03DB01, R03DB05, R05CA10, R05CB10, R05DA, R05DB, R05FA01, R05FA02, R05FB02, R05X, R06AA, R06AA02, R06AA04, R06AA08, R06AA09, R06AA52, R06AB01, R06AB02, R06AB04, R06AB54, R06AD02, R06AE05, R06AE55, R06AK, R06AX02, S01FA01, S01GX99

© 2020 Huang W-K et al. *JAMA Network Open*.

eTable 3. Crude and Adjusted Hazard Ratios for the Association Between Antiandrogen Monotherapy and All-Cause Dementia by Cumulative Use After Prostate Cancer Diagnosis

	**Crude**			**Stabilize IPTW**			**Multivariate Cox[a]**	
	HR (95% CI)	p		HR (95% CI)	p		HR (95% CI)	p
**Nonuser**	1.00 (reference)			1.00 (reference)			1.00 (reference)	
**0-1 yrs**	2.10 (1.79-2.46)	<.0001		1.36 (1.16-1.60)	0.0002		1.47 (1.25-1.73)	<.0001
**1-2 yrs**	2.42 (1.93-3.02)	<.0001		1.63 (1.28-2.07)	<.0001		1.33 (1.06-1.68)	0.0142
**2-3 yrs**	1.27 (0.86-1.88)	0.2326		0.88 (0.58-1.34)	0.5592		0.80 (0.54-1.19)	0.2654
**>3 yrs**	1.06 (0.63-1.78)	0.8299		0.75 (0.43-1.29)	0.2953		0.74 (0.44-1.24)	0.2526

Abbreviations: HR, Hazard ratio; IPTW, inverse probability treatment weighting.

[a] Age as the time scale. Adjusted for urbanization, monthly income, enrollee category, comorbidities (listed in table 1), benzodiaepine, and anti-cholinergic use, and number of clinical visits.

© 2020 Huang W-K et al. *JAMA Network Open*.
